# New‐onset myasthenia gravis after novel coronavirus 2019 infection

**DOI:** 10.1002/rcr2.978

**Published:** 2022-05-22

**Authors:** Amirmasoud Taheri, Lotfollah Davoodi, Eissa Soleymani, Noushin Ahmadi

**Affiliations:** ^1^ Mazandaran University of Medical Sciences Sari Iran; ^2^ Antimicrobial Resistance Research Center, Communicable Diseases Institute, and Department of Infectious Diseases Mazandaran University of Medical Sciences Sari Iran; ^3^ Parasitology, Hamadan University of Medical Sciences Hamadan Iran

**Keywords:** COVID‐19, diplopia, myasthenia gravis, neuromuscular disorders

## Abstract

In the years since the start of the COVID‐19 pandemic, numerous neurological manifestations have been reported following this novel virus. Myasthenia gravis is one of them. Here, we present the patient that was referred to us with myasthenia gravis symptoms after a COVID‐19 infection.

## Graphical abstract

Myasthenia gravis (MG) is an autoimmune disease in which the neuromuscular junction is attacked by multiple autoantibodies. To date, few cases of post‐COVID new onset ocular MG have been reported; here we present a generalized MG after COVID‐19 infection.

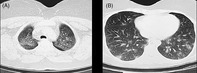

## INTRODUCTION

In late December 2019, the world was confronted with a new challenge: coronavirus disease 2019 (COVID‐19). A new illness caused by the severe acute respiratory syndrome coronavirus 2 (SARS‐CoV‐2) that was first reported in Wuhan, China. Up to now, the World Health Organization has reported about 390 million confirmed cases and 5.7 million deaths (WHO) due to COVID‐19.^1^ Besides the lungs, this virus can cause multi‐organ failure.^2^ Additionally, it can also affect the nervous system. Several neurological involvements have been reported, including anosmia/ageusia, stroke, Guillain‐Barré‐Syndrome, Miller‐Fisher‐Syndrome, neuropathy, myopathy, neuromuscular disorders.^3^

Myasthenia gravis (MG) is an autoimmune disease in which the neuromuscular junction is attacked by multiple autoantibodies. It can cause weakness in any skeletal muscle group, but it is usually associated with classical symptoms, such as ptosis and/or diplopia in the eyes, as well as bulbar symptoms including dysarthria, dysphagia, and fatigable chewing.^4^ To date, few cases of post‐COVID new onset ocular MG have been reported^5^, ^6^, ^7^; here we present a generalized MG after COVID‐19 infection.

## CASE REPORT

**FIGURE 1:**
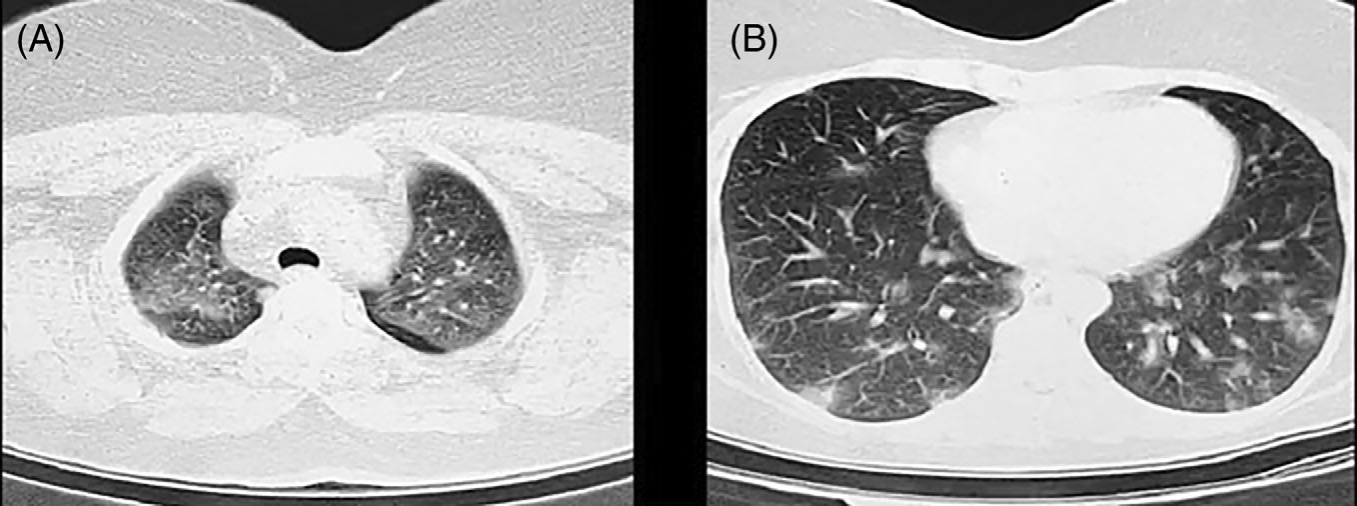
An axial section of the chest computed tomography [CT] scan. Ground glass opacities (A) and peripheral consolidation (B)

In early September 2021, a 35‐year‐old woman was referred to the emergency department (ED) of the Razi hospital, Qaemshahr, northern Iran with a complaint of dyspnea, myalgia and the sore throat from a week ago. She also complained of weakness, nausea, and coughing. There were no underlying diseases in her or her parents. At ED triage, they recorded a blood pressure of 135/85 mmHg, a heart rate of 106 beats/min, a respiratory rate of 26 breaths per minute, a temperature of 38.9°C, and an oxygen saturation (SpO_2_) of 94%. A physical examination revealed nothing unusual except for fine crackles in both lungs. A severe acute respiratory syndrome coronavirus 2 (SARS‐CoV‐2) PCR test was performed for the patient at the ED triage, which revealed a positive result. Due to her signs and symptoms, plus a positive PCR test, a chest CT‐scan was requested. As demonstrated in Figure 1, peripheral consolidation and ground glass opacity were seen in her chest CT‐scan. Over the next 6 days, dexamethasone 8 mg/day, remdesivir 200 mg on the first day and 100 mg/day for the next 5 days, and medications for accompanying symptoms like cough, fever, etc. were given. After 6 days of admission, the patient was discharged in a healthy condition with normal vital signs.

She returned to our ED 2 weeks after discharge. This time she complained of severe weakness in her upper and lower limbs, and blurred vision and droopy eyelids, which started 3 days ago. Her vital signs and SpO_2_ were normal. A neurological examination showed ptosis of both eyelids, and all four limbs had a grade 3 weakness on the Medical Research Council scale (movement against gravity is possible but not against resistance of the examiner, grade 0 (lowest) = no movement, grade 5 (highest) = muscle contracts normally). Examinations of other organs, particularly the respiratory system, were normal. Her signs and symptoms led us to suspect MG, so an ice test was performed, which revealed a remarkable improvement in ptosis. Following consultation with a neurologist, specific diagnostic serologic test and single‐fibre electromyography (SFEMG) were performed. Apart from high levels of acetylcholine receptor antibodies (0.57 nmol/L with the normal range of >0.45 nmol/L) other lab tests were normal. SFEMG demonstrated an increased jitter (action potential threshold to endplate potential ratio larger than normal), with blockade (inability to elicit muscle fibre due to impaired neuromuscular transmission) in more than two pairs of orbicularis oculi muscle. Thymoma was ruled out after a review of her earlier chest CT scan. The patient was treated with pyridostigmine 60 mg three times a day for as long as she is symptomatic. During her weekly follow‐up reviews, her symptoms steadily improved.

## DISCUSSION

In this case report, we present a young woman with class IIIa MG according to the Myasthenia Gravis Foundation of America (MGFA) classification. MG is an autoimmune disease that is mainly caused by antibodies against the acetylcholine receptor.^4^ One of the contentious mechanisms for inducing autoantibody production is viral infection.^8^ There have been a few reports of post‐infectious MG [post varicella,^9^ post pharyngitis,^10^ post Epstein–Barr virus (EBV)^11^]. Up to now, several case reports of COVID‐19 infection in pre‐existing MG,^12^, ^13^, ^14^ and also new onset post‐COVID‐19 MG have been reported.^5^, ^6^, ^7^ The exact mechanism is not fully known, but post‐viral autoantibody production due to COVID‐19 has been speculated.^15^, ^16^

To the best of our knowledge, our patient is the 5th post‐COVID‐19 MG that has been reported. As the number of this rare presentation rises, there is a greater need for more in‐depth research into this disease's pathophysiology.

## AUTHOR CONTRIBUTIONS

Lotfollah Davoodi, Noushin Ahmadi involved in the clarification and collecting of data. Amirmasoud Taheri, Lotfollah Davoodi involved in writing of the manuscript draft. Amirmasoud Taheri, Lotfollah Davoodi and Eissa Soleymani involved in editing of the manuscript. Amirmasoud Taheri, Lotfollah Davoodi is involved in critically revising the whole manuscript. Amirmasoud Taheri and Lotfollah Davoodi are responsible for presenting data and submitting the manuscript. All authors reviewed and approved the final version of the manuscript.

## CONFLICT OF INTEREST

None declared.

## ETHICS STATEMENT

The authors declare that appropriate written informed consent was obtained for the publication of this manuscript and accompanying images.

## Data Availability

The data are available with the correspondence author and can be reached on request.
